# Relationship between epa level of supervision with their associated subcompetency milestone levels in pediatric fellow assessment

**DOI:** 10.1186/s12909-023-04689-0

**Published:** 2023-10-03

**Authors:** Richard B. Mink, Carol L. Carraccio, Bruce E. Herman, Pnina Weiss, David A. Turner, Diane E. J. Stafford, Kathleen A. McGann, Jennifer Kesselheim, Deborah C. Hsu, Pamela C. High, Jill J. Fussell, Megan L. Curran, Patricia R. Chess, Cary Sauer, Sarah Pitts, Angela L. Myers, John D. Mahan, Christiane E. L. Dammann, Tandy Aye, Alan Schwartz, for the Subspecialty Pediatrics Investigator Network, Vinod Havalad, Joaquim Pinheiro, Elizabeth Alderman, Mamta Fuloria, Megan E. McCabe, Jay Mehta, Yolanda Rivas, Maris Rosenberg, Cara Doughty, Albert Hergenroeder, Arundhati Kale, YoungNa Lee-Kim, Jennifer A. Rama, Phil Steuber, Bob Voigt, Karen Hardy, Samantha Johnston, Debra Boyer, Carrie Mauras, Alison Schonwald, Tanvi Sharma, Christine Barron, Penny Dennehy, Elizabeth S. Jacobs, Jennifer Welch, Deepak Kumar, Katherine Mason, Nancy Roizen, Jerri A. Rose, Brooke Bokor, Jennifer I. Chapman, Lowell Frank, Iman Sami, Jennifer Schuette, Ramona E. Lutes, Stephanie Savelli, Rambod Amirnovin, Rula Harb, Roberta Kato, Karen Marzan, Roshanak Monzavi, Doug Vanderbilt, Lesley Doughty, Constance McAneney, Ward Rice, Lea Widdice, Fran Erenberg, Blanca E. Gonzalez, Deanna Adkins, Deanna Green, Aditee Narayan, Kyle Rehder, Joel Clingenpeel, Suzanne Starling, Heidi Eigenrauch Karpen, Kelly Rouster-Stevens, Jatinder Bhatia, John Fuqua, Jennifer Anders, Maria Trent, Rangasamy Ramanathan, Yona Nicolau, Allen J. Dozor, Thomas Bernard Kinane, Takara Stanley, Amulya Nageswara Rao, Meredith Bone, Lauren Camarda, Viday Heffner, Olivia Kim, Jay Nocton, Angela L. Rabbitt, Richard Tower, Michelle Amaya, Jennifer Jaroscak, James Kiger, Michelle Macias, Olivia Titus, Modupe Awonuga, Karen Vogt, Anne Warwick, Dan Coury, Mark Hall, Megan Letson, Melissa Rose, Julie Glickstein, Sarah Lusman, Cindy Roskind, Karen Soren, Jason Katz, Lorena Siqueira, Mark Atlas, Andrew Blaufox, Beth Gottleib, David Meryash, Patricia Vuguin, Toba Weinstein, Laurie Armsby, Lisa Madison, Brian Scottoline, Evan Shereck, Michael Henry, Patricia A. Teaford, Sarah Long, Laurie Varlotta, Alan Zubrow, Courtenay Barlow, Heidi Feldman, Hayley Ganz, Paul Grimm, Tzielan Lee, Leonard B. Weiner, Zarela Molle-Rios, Nicholas Slamon, Ursula Guillen, Karen Miller, Myke Federman, Randy Cron, Wyn Hoover, Tina Simpson, Margaret Winkler, Nada Harik, Ashley Ross, Omar Al-Ibrahim, Frank P. Carnevale, Wayne Waz, Fayez Bany-Mohammed, Jae H. Kim, Beth Printz, Mike Brook, Michelle Hermiston, Erica Lawson, Sandrijn van Schaik, Alisa McQueen, Karin Vander Ploeg Booth, Melissa Tesher, Jennifer Barker, Sandra Friedman, Ricky Mohon, Andrew Sirotnak, John Brancato, Wael N. Sayej, Nizar Maraqa, Michael Haller, Brenda Stryjewski, Pat Brophy, Riad Rahhal, Ben Reinking, Paige Volk, Kristina Bryant, Melissa Currie, Katherine Potter, Alison Falck, Joel Weiner, Michele M. Carney, Barbara Felt, Andy Barnes, Catherine M. Bendel, Bryce Binstadt, Karina Carlson, Carol Garrison, Mary Moffatt, John Rosen, Jotishna Sharma, Kelly S. Tieves, Hao Hsu, John Kugler, Kari Simonsen, Rebecca K. Fastle, Doug Dannaway, Sowmya Krishnan, Laura McGuinn, Mark Lowe, Selma Feldman Witchel, Loreta Matheo, Rebecca Abell, Mary Caserta, Emily Nazarian, Susan Yussman, Alicia Diaz Thomas, David S. Hains, Ajay J. Talati, Elisabeth Adderson, Nancy Kellogg, Margarita Vasquez, Coburn Allen, Luc P. Brion, Michael Green, Janna Journeycake, Kenneth Yen, Ray Quigley, Anne Blaschke, Susan L. Bratton, Christian Con Yost, Susan P. Etheridge, Toni Laskey, John Pohl, Joyce Soprano, Karen Fairchild, Vicky Norwood, Troy Alan Johnston, Eileen Klein, Matthew Kronman, Kabita Nanda, Lincoln Smith, David Allen, John G. Frohna, Neha Patel, Cristina Estrada, Geoffrey M. Fleming, Maria Gillam-Krakauer, Paul Moore, Joseph Chaker El-Khoury, Jennifer Helderman, Greg Barretto, Kelly Levasseur, Lindsay Johnston

**Affiliations:** 1 Department of Pediatrics, David Geffen School of Medicine at UCLA and the Lundquist Institute for Biomedical Innovation at Harbor-UCLA Medical Center, 1124 West Carson Street, Torrance, CA 90502 USA; 2 American Board of Pediatrics, Chapel Hill, NC USA; 3 University of Utah School of Medicine, Salt Lake, UT USA; 4 Department of Pediatrics, Yale School of Medicine, New Haven, CT USA; 5 Division of Endocrinology, Department of Pediatrics, Stanford University School of Medicine, Palo Alto, CA USA; 6 Department of Pediatrics, Duke University Medical Center, Durham, NC USA; 7 Dana-Farber/Boston Children’s Cancer and Blood Disorders Center, Boston, MA USA; 8 Baylor College of Medicine, Houston, TX USA; 9 Alpert Medical School of Brown University, Providence, RI USA; 10 Developmental-Behavioral Pediatrics, Hasbro Children’s Hospital, Providence, RI USA; 11 University of Arkansas for Medical Sciences and Arkansas Children’s Hospital, Little Rock, AR USA; 12 Department of Pediatrics, University of Colorado School of Medicine, Aurora, CO USA; 13 University of Rochester, Rochester, NY USA; 14 Department of Pediatrics, Emory University School of Medicine and Children’s Healthcare of Atlanta, Atlanta, GA USA; 15 Division of Adolescent/Young Adult Medicine, Boston Children’s Hospital, Boston, MA USA; 16 Center for Wellbeing, Children’s Mercy Hospital and University of Missouri-Kansas City School of Medicine, Kansas City, MO USA; 17 Department of Pediatrics, Nationwide Children’s Hospital and The Ohio State University College of Medicine, Columbus, OH USA; 18 Tufts Children’s Hospital, Tufts University, Boston, MA USA; 19 University of Illinois College of Medicine at Chicago, Chicago, IL USA

**Keywords:** EPAs, Milestones, Assessment, Correlation, Fellow, Equations

## Abstract

**Background:**

Entrustable Professional Activities (EPA) and competencies represent components of a competency-based education framework. EPAs are assessed based on the level of supervision (LOS) necessary to perform the activity safely and effectively. The broad competencies, broken down into narrower subcompetencies, are assessed using milestones, observable behaviors of one’s abilities along a developmental spectrum. Integration of the two methods, accomplished by mapping the most relevant subcompetencies to each EPA, may provide a cross check between the two forms of assessment and uncover those subcompetencies that have the greatest influence on the EPA assessment.

**Objectives:**

We hypothesized that 1) there would be a strong correlation between EPA LOS ratings with the milestone levels for the subcompetencies mapped to the EPA; 2) some subcompetencies would be more critical in determining entrustment decisions than others, and 3) the correlation would be weaker if the analysis included only milestones reported to the Accreditation Council for Graduate Medical Education (ACGME).

**Methods:**

In fall 2014 and spring 2015, the Subspecialty Pediatrics Investigator Network asked Clinical Competency Committees to assign milestone levels to each trainee enrolled in a pediatric fellowship for all subcompetencies mapped to 6 Common Pediatric Subspecialty EPAs as well as provide a rating for each EPA based upon a 5-point LOS scale.

**Results:**

One-thousand forty fellows were assessed in fall and 1048 in spring, representing about 27% of all fellows. For each EPA and in both periods, the average milestone level was highly correlated with LOS (rho range 0.59–0.74; *p* < 0.001). Correlations were similar when using a weighted versus unweighted milestone score or using only the ACGME reported milestones (*p* > 0.05).

**Conclusions:**

We found a strong relationship between milestone level and EPA LOS rating but no difference if the subcompetencies were weighted, or if only milestones reported to the ACGME were used. Our results suggest that representative behaviors needed to effectively perform the EPA, such as key subcompetencies and milestones, allow for future language adaptations while still supporting the current model of assessment. In addition, these data provide additional validity evidence for using these complementary tools in building a program of assessment.

## Background

Early in the transition to a competency-based model for trainee education and assessment, identifying the Accreditation Council for Graduate Medical Education (ACGME) core competencies in the United States (US) and the CanMeds roles in Canada were critical first steps [1, 2]. Each of the core competencies was further refined into specific “subcompetencies” in the US and each CanMeds role elaborated and defined. An important next step was the creation of milestones specific to the subcompetency or the role [3, 4]. The milestones represent defined, observable abilities of an individual’s skills along a developmental continuum [5]. In the US, each specialty was tasked with creating both the subcompetencies and milestones for the ACGME competencies [3]. Pediatrics created milestones for 48 subcompetencies, of which only 21 were reported to the Accreditation Council for Graduate Medical Education (ACGME) biannually for all trainees [6]. Milestone ratings ranged from one to four or one to five, but trainees were not necessarily expected to achieve the highest levels at the time of graduation.

The subsequent creation of Entrustable Professional Activities (EPAs) by ten Cate and Scheele [7] complements the milestones by providing a meaningful clinical context for the subcompetencies. EPAs are observable activities of a profession that an individual should be able to execute without supervision when in practice [8–11]. As opposed to subcompetencies, EPA assessments are based upon the amount of supervision a trainee needs to perform the activity safely and effectively, ranging from direct to indirect to none [12, 13]. Basing EPA judgements on needed levels of trainee supervision aligns what faculty do in real time with what they are asked to do as part of the assessment process, thus adding to their validity evidence [14].

**Fig. 1:**
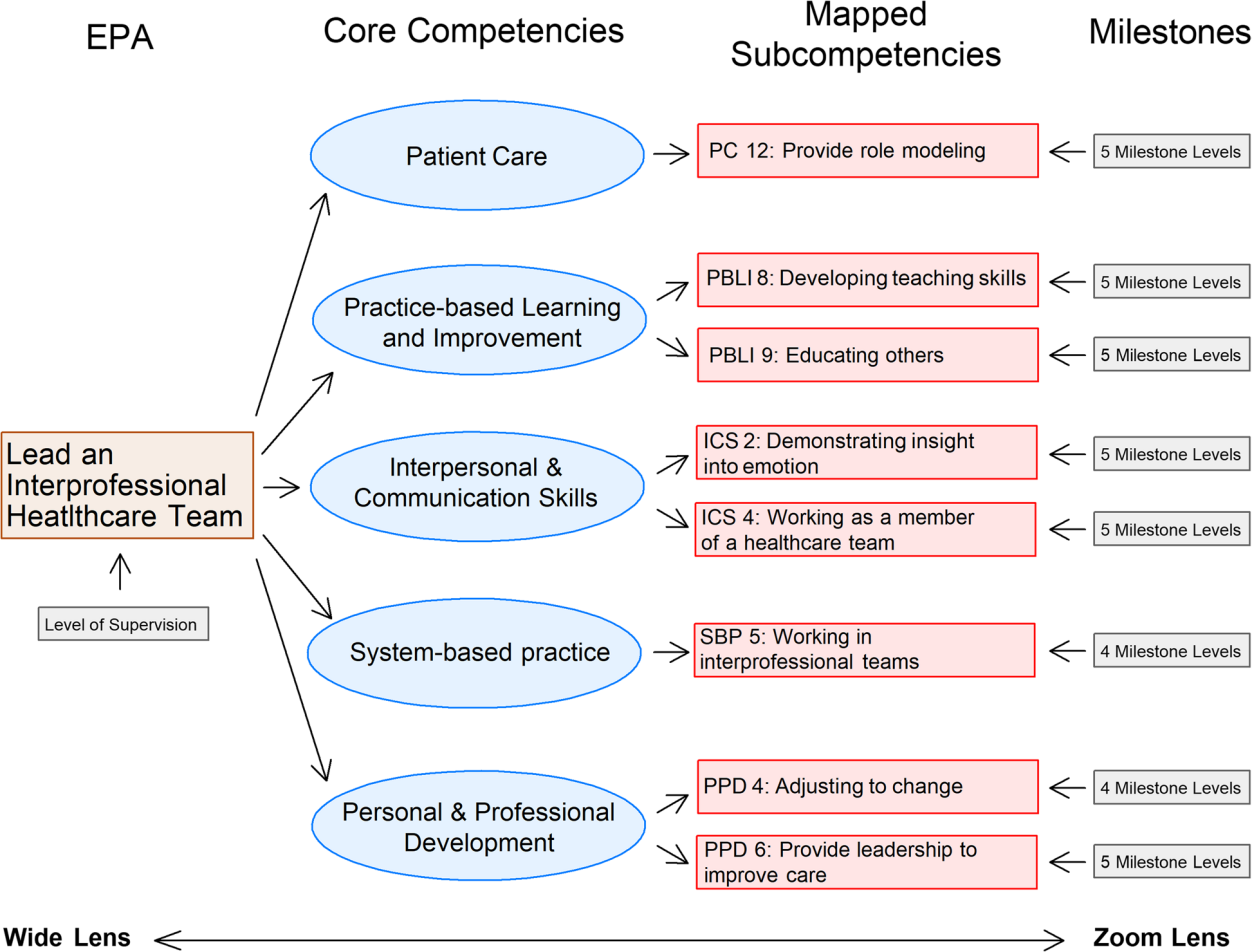
Schematic showing the relationship of the Lead an Interprofessional Healthcare Team EPA with mapped core competencies and subcompetencies. Eight subcompetencies map to this EPA. Milestones were created for all subcompetencies but of the 8, only 5 are reported to the Accreditation Council for Graduate Medical Education. Personal & Professional Development (PPD) is a core competency unique to Pediatrics. Abbreviations: PC = patient care, PBLI = practice-based learning and improvement, ICS = interpersonal and communication skills, SBP = system-based practice

To link EPA and milestone assessments, medical education leaders then mapped the subcompetencies thought to be critical in executing the professional activities of each EPA [7, 15, 16]. An example of the mapping of the Leadteam EPA (Table 1) is illustrated in Fig. 1. For this EPA, 8 subcompetencies were judged to be important in making the entrustment decision. Milestones for 5 of the 8 subcompetencies are required to be reported to the ACGME in the fall and spring each year. While mapping was accomplished by experts through an iterative process, data supporting the mapping are lacking and it is unknown if any specific subcompetency is more important than the others in making the entrustment decision. Similarly, it is unclear whether using all the mapped subcompetencies, or only those required to be reported to the ACGME, are critical in formulating the entrustment decision. This information would be helpful to know for future studies, particularly if the milestone levels could be obtained directly from the ACGME.

**Table 1:** The six Common Pediatric Subspecialty EPAs evaluated in this study with their abbreviations and scales for EPA level of supervision

Common Pediatric Subspecialty EPA	Abbreviation
***Apply public health principles and quality improvement methods to improve population health***	**QI**
Level 1: Trusted to observe only	
Level 2: Trusted to contribute with direct supervision and coaching as a member of a collaborative effort to improve care at the institutional level	
Level 3: Trusted to contribute without direct coaching as a member of a collaborative effort to improve care at the institutional level	
Level 4: Trusted to lead collaborative efforts to improve care for populations and systems at the institutional level	
Level 5: Trusted to lead collaborative efforts to improve care at the level of populations and systems at the regional and/or national level	
***Provide consultation to other healthcare providers caring for children and adolescents and refer patients requiring further consultation to other subspecialty providers if necessary***	**Consultation**
Level 1:Trusted to observe only	
Level 2:Trusted to execute with direct supervision and coaching	
Level 3:Trusted to execute with indirect supervision and discussion of information conveyed for most simple and some complex cases	
Level 4:Trusted to execute with indirect supervision and may require discussion of information conveyed but only for selected complex cases	
Level 5:Trusted to execute independently without supervision	
***Contribute to the fiscally sound, equitable and collaborative management of a healthcare workplace***	**Management**
Level 1:Trusted to observe only	
Level 2:Trusted to perform with direct supervision and coaching with supervisor verifying work product for accuracy	
Level 3:Trusted to perform with supervisor serving as a consultant for all tasks	
Level 4:Trusted to perform with supervisor serving as a consultant but only for complex tasks	
Level 5:Trusted to perform without supervision	
***Facilitate handovers to another healthcare provider either within or across settings***	**Handover**
Level 1:Trusted to observe only	
Level 2:Trusted to execute with direct supervision and coaching	
Level 3:Trusted to execute with indirect supervision with verification of information after the handover for most simple and some complex cases	
Level 4:Trusted to execute with indirect supervision with verification of information after the handover for selected complex cases	
Level 5:Trusted to execute without supervision	
***Lead an interprofessional health care team***	**Leadteam**
Level 1:Trusted to participate only	
Level 2:Trusted to lead with direct supervision and coaching	
Level 3:Trusted to lead with supervisor occasionally present to provide advice	
Level 4:Trusted to lead without supervisor present but requires coaching to improve member and team performance	
Level 5:Trusted to lead without supervision to improve member and team performance	
***Lead within the subspecialty profession***	**Leadprof**
Level 1:Trusted to observe only	
Level 2:Trusted to contribute to advocacy and public education activities for the subspecialty profession with direct supervision and coaching at the institutional level	
Level 3:Trusted to contribute to advocacy and public education activities for the subspecialty profession with indirect supervision at the institutional level	
Level 4:Trusted to mentor others and lead advocacy and public education activities for the subspecialty profession at the institutional level	
Level 5:Trusted to lead advocacy and public education activities for the subspecialty profession at the regional and/or national level

Milestones and EPA level of supervision (LOS) both represent elements of a system of trainee assessment [17]. While all ACGME accredited programs must report milestones, many specialties are now promoting the use of EPA LOS for assessment. The American Board of Pediatrics announced that it will begin using EPA LOS ratings to determine eligibility to sit for its certification exams [18]. In July 2023, the American Board of Surgery began using EPAs as the foundation for competency-based surgical training [19]. Both Emergency Medicine and Family Medicine, along with other disciplines, are also currently exploring the use of EPA LOS in their training programs [18]. Since EPAs provide a holistic view (“wide lens” in Fig. 1) of the execution of an activity, and milestones provide a more granular assessment (“narrow lens”) of specific behaviors needed to perform them, there should be a strong relationship between these two approaches to assessment [20]. This relationship requires further exploration as the finding of a strong association between the two would provide validity evidence for both types of assessments.

Using this logic, our hypotheses were: 1) there is a strong correlation between EPA LOS rating with the average score of the mapped subcompetency milestone levels needed to perform the EPA; 2) some subcompetencies would be more critical than others such that weighted scores will have a stronger correlation; and 3) if only those milestones required for reporting to the ACGME were included in the analysis, the correlation between EPA LOS rating and average milestone level would be weaker.

## Methods

We performed the study using the Subspecialty Pediatrics Investigator Network (SPIN), a medical education research network that includes representatives from each of the 14 pediatric subspecialties with primary American Board of Pediatrics certification as well as the Council of Pediatric Subspecialties, Association of Pediatric Program Directors Fellowship Executive Committee, and Association of Pediatric Program Directors Longitudinal Educational Assessment Research Network (APPD LEARN) [21]. The goal was to recruit at least 20% of fellowships from each subspecialty. We obtained IRB approval from each participating institution with the University of Utah serving as the lead.

One week before the Clinical Competency Committee (CCC) meeting, we asked fellowship program directors (FPDs) to assign a LOS rating for each fellow for 6 of the 7 EPAs common to all pediatric subspecialties (Common Pediatric Subspecialty EPAs; Table 1) [22]. Then, at the CCC meeting, we asked the members to first assign a milestone level to all 29 subcompetencies mapped to these six EPAs. Of note, in Pediatrics, all subspecialties utilize the same milestones. CCCs then assigned a LOS rating for each fellow for each Common Pediatric Subspecialty EPA. We provided no specific instructions to the FPD or CCC members about the procedure to determine fellow ratings or faculty development about EPAs or the EPA LOS scales. Representatives of the 14 subspecialties contributed to the development of the EPA LOS scales and the validity evidence for them has previously been published [23, 24]. Designed to be intuitive, these ordinal 5-level scales are based upon direct, indirect and no supervision with case complexity being a variable in determining the need for supervision at some levels for some EPAs (Table 1).

The anonymity of trainees was ensured by creating a unique participant identifier number using an algorithm developed by APPD LEARN [25]. Once this ID was created, we provided specific links to the online data collection instruments. In the survey instrument, we first elicited milestone ratings for each of the subcompetencies grouped by the 6 core competencies and then obtained LOS ratings for each EPA. When presenting the subcompetency, we displayed the subcompetency name and descriptions for each milestone; when presenting each EPA, we displayed the title of the EPA and the associated functions necessary to carry out the activities followed by the LOS scale.

We also collected information about each fellow’s subspecialty and year of fellowship, institution, the number of fellows in the program, how long the FPD served in this role, and FPDs self-reported understanding of EPAs (unfamiliar, basic, in-depth, or expert). We also asked whether the FPD was a member of the CCC since FPD participation on the CCC may influence assignment of trainee ratings [24]. Details about the data collection tools have been previously described [23]. We collected data in fall 2014 and spring 2015. The abbreviations for each EPA are listed in Table 1.

For each EPA, we computed an unweighted composite milestone score by averaging the milestone levels for the subcompetencies mapped to that EPA. We compared LOS ratings and unweighted composite score for trainees at each data collection period using linear mixed models adjusting for repeated measures and clustering within programs.

We computed a weighted composite milestone score for each EPA by using a confirmatory factor analysis procedure to fit path coefficients and mean structures between each EPA’s LOS and its mapped subcompetencies, adjusting for clustering in program, and then used the path coefficients to generate a weighted average of the milestone levels. To assess the fit of the procedure, we examined the comparative fit index and the root mean squared error of approximation using the entire sample; to guard against overfitting, we also conducted a fivefold cross-validation bootstrap process, fitting the path coefficients on 80% of the data and making predictions on the remaining 20%, repeating the process for each fold and averaging 500 replications.

We tested the hypothesis that composite milestone scores would be correlated with LOS ratings using Spearman’s ρ. We tested the hypothesis that weighted composites would outperform unweighted composites by comparing confidence intervals around the ρ values for the weighted and unweighted composites, and similarly tested differences between unweighted composites at programs where the FPD did or did not serve on the CCC. We tested the hypothesis that using all critical subcompetencies would better predict LOS than using only ACGME-reported milestones in a similar fashion, and directly compared the fit of the nested weighted confirmatory factor analysis models using a likelihood ratio χ^2^ test.

We generated equations to predict milestone levels using the path coefficients in the confirmatory factor analysis for each model. For external validation of the predicted levels, we used spring 2019 EPA LOS ratings that were obtained in a recently completed study [26]. EPA LOS ratings were collected in the same manner as described above except that milestone levels were not obtained. Spring 2019 milestone levels were provided by the ACGME through a data sharing agreement with APPD LEARN. With these data, we examined the goodness-of-fit using the ACGME equations.

Using the model with the best fit and parsimony, we constructed receiver operating characteristic (ROC) curves for the ability of that model’s composite milestone score to discriminate between decisions affirming or refuting entrustment, using levels 4 or 5 as the minimum level for affirmation of entrustment. Data analyses were conducted using R 3.6 (R Core Team, Vienna, Austria).

## Results

We assessed 1040 fellows in fall and 1048 in spring, representing about 27% of all pediatric fellows [27] Data were submitted from 78 and 82 different institutions and 209 and 212 programs in fall and spring, respectively. In both periods, 79% (11/14) of subspecialties met our goal of having at least 20% of their subspecialty programs provide data. FPDs were a member of the CCC for 57.5% (598/1040) of ratings in the first data collection period and 55.7% (584/1048) in the second. FPDs completed their assessments a median of 6[IQR 1–9] and 7 [1-11] days before the CCC meeting in fall and spring, respectively.

Mean EPA LOS and unweighted composite milestone scores for each period are displayed in Table 2. Both EPA LOS and milestone score increased from the fall to the spring (*p* < 0.001 for each EPA, adjusting for repeated measures and clustering of trainees in programs).

**Table 2:** Overall mean (95% CI) unweighted composite subcompetency milestone scores and EPA level of supervision ratings in fall 2014 and spring 2015

EPA	Period	Unweighted composite milestone score^a^	Mean EPA LOS Rating^a^
QI	Fall	3.1 (3.1–3.1)	2.7 (2.6–2.7)
	Spring	3.3 (3.3–3.4)	3.0 (2.9–3.0)
Consultation	Fall	3.3 (3.3–3.3)	3.4 (3.4–3.5)
	Spring	3.5 (3.5–3.6)	3.8 (3.7–3.8)
Management	Fall	3.2 (3.2–3.2)	2.8 (2.7–2.8)
	Spring	3.5 (3.5–3.5)	3.2 (3.1–3.2)
Handover	Fall	3.3 (3.3–3.4)	3.7 (3.6–3.7)
	Spring	3.6 (3.6–3.6)	4.0 (4.0–4.1)
Leadteam	Fall	3.3 (3.2–3.3)	3.0 (3.0–3.0)
	Spring	3.5 (3.5–3.5)	3.4 (3.3–3.4)
Leadprof	Fall	3.2 (3.1–3.2)	2.7 (2.6–2.7)
	Spring	3.4 (3.4–3.4)	3.0 (3.0–3.1)

*Abbreviations*: *EPA* Entrustable professional activities, *LOS* level of supervision

^a^*p* < 0.05 fall vs. spring for all EPAs

### Testing hypothesis 1: There is a strong correlation between EPA LOS rating with the average score of the mapped subcompetency milestone levels needed to perform the EPA

**Fig. 2:**
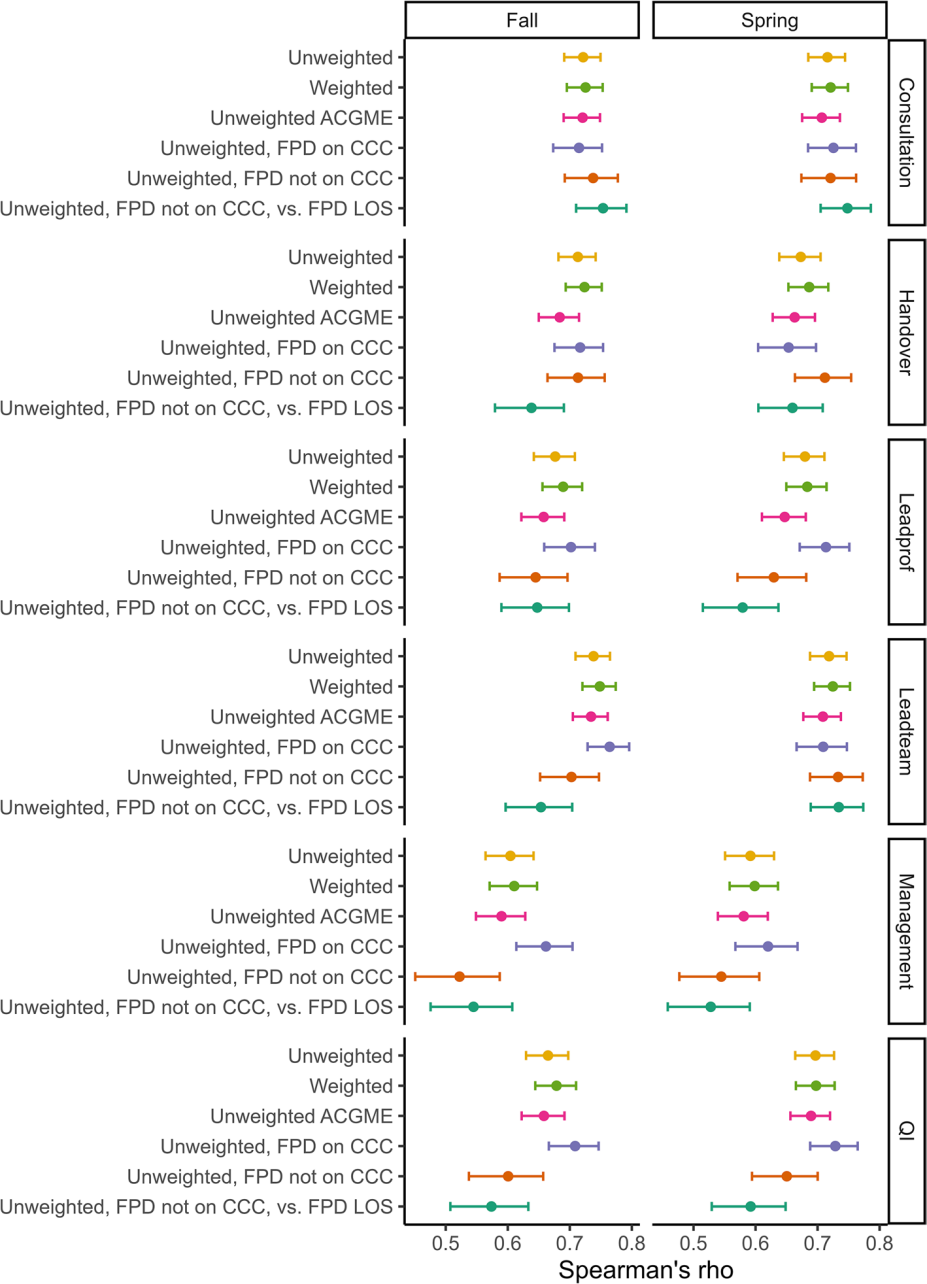
Graph showing Spearman Rho correlations (95% confidence intervals) of EPA level of supervision ratings by the Clinical Competency Committee with unweighted (#1) and weighted (#2) composite score using all mapped milestones, unweighted using only the ACGME reported milestones (#3) and unweighted using milestones from when the fellowship program director was (#4) or was not (#5) a member of the Clinical Competency Committee. The last graph in each group (#6) shows the correlation of EPA level of supervision ratings made independently by the fellowship program director with milestones for when the program director was not a member of the clinical competency committee. Data are from the fall 2014 and spring 2015. Abbreviations: ACGME = Accreditation Council for Graduate Medical Education, CCC = Clinical Competency Committee, EPA = Entrustable professional activities, FPD = fellowship program director; LOS = level of supervision

There was moderate to strong correlation between the unweighted composite milestone score and EPA LOS, ranging from 0.59 for the Management EPA to 0.74 for Leadteam, supporting our first hypothesis (Fig. 2, Table 3). There was no difference in the correlations between the two periods (*p* > 0.05). Correlations between LOS ratings made independently by the FPD before the CCC meeting with composite milestone scores were similar to those made by the CCC when the FPD was not a member. In addition, when examining the associations in programs where the FPD was not a CCC member versus where the FPD was a member, the correlations were somewhat lower for some EPAs (QI and Management in the fall). Otherwise, they were not significantly different. The significant associations between milestone and EPA LOS ratings persisted after adjustment for institution, subspecialty, and program and FPD characteristics.

**Table 3:** Correlation [Rho (95% CI)] of the unweighted and weighted composite subcompetency milestone score with EPA level of supervision ratings in fall 2014 and spring 2015 using all milestone data, only ACGME reported milestones and whether the program director was or was not a member of Clinical Competency Committee

Source of Data		All CCC members	All CCC members	All CCC members, only ACGME reported milestones	FPD on CCC	FPD not on CCC	FPD not on CCC
**EPA**	**Period**	**Unweighted composite milestone score vs CCC LOS**^a^	**Weighted composite milestone score vs CCC LOS**^a^	**Unweighted composite milestone score vs CCC LOS**^a^	**Unweighted composite milestone score vs CCC LOS**^a^	**Unweighted composite milestone score vs CCC LOS**^**a**^	**Unweighted composite milestone score vs FPD LOS**^a^
**QI**	Fall	0.66(0.63—0.70)	0.68(0.64–0.71)	0.66(0.62–0.69)	0.71(0.67–0.75)	0.60(0.54–0.66)^b^	0.57(0.51–0.63)^c^
	Spring	0.70(0.66—0.73)	0.70(0.67–0.73)	0.69(0.66–0.72)	0.73(0.69–0.76)	0.65(0.59–0.70)	0.59(0.53–0.65)^c^
**Consultation**	Fall	0.72(0.69—0.75)	0.73(0.70–0.75)	0.72(0.69–0.75)	0.71(0.67–0.75)	0.74(0.69–0.78)	0.75(0.71–0.79)
	Spring	0.72(0.69—0.74)	0.72(0.69–0.75)	0.71(0.68–0.74)	0.73(0.68–0.76)	0.72(0.67–0.76)	0.75(0.70–0.79)
**Management**	Fall	0.60(0.56—0.64)	0.61(0.57–0.65)	0.59(0.55–0.63)	0.66(0.61–0.70)	0.52(0.45–0.59)^b^	0.54(0.48–0.61)
	Spring	0.59(0.55—0.63)	0.60(0.56–0.64)	0.58(0.54–0.62)	0.62(0.57–0.67)	0.54(0.47–0.61)	0.53(0.46–0.59)
**Handover**	Fall	0.71(0.68—0.74)	0.72(0.69–0.75)	0.68(0.65–0.71)	0.72(0.68–0.75)	0.71(0.66–0.76)	0.64(0.58–0.69)
	Spring	0.67(0.64—0.71)	0.69(0.65–0.72)	0.66(0.63–0.70)	0.65(0.60–0.70)	0.71(0.66–0.75)	0.66(0.60–0.71)
**Leadteam**	Fall	0.74(0.71—0.76)	0.75(0.72–0.77)	0.73(0.70–0.76)	0.76(0.73–0.80)	0.70(0.65–0.75)	0.65(0.60–0.70)^c^
	Spring	0.72(0.68—0.75)	0.72(0.69–0.75)	0.71(0.68–0.74)	0.71(0.67–0.75)	0.73(0.69–0.77)	0.73(0.69–0.77)
**Leadprof**	Fall	0.68(0.64—0.71)	0.69(0.66–0.72)	0.66(0.62–0.69)	0.70(0.66–0.74)	0.64(0.59–0.70)	0.65(0.59–0.70)
	Spring	0.68(0.64—0.71)	0.68(0.65–0.71)	0.65(0.61–0.68)	0.71(0.67–0.75)	0.63(0.57–0.68)	0.58(0.52–0.64)^c^

^a^all values *p* < 0.001; ^b^*p* < 0.05 compared with unweighted composite milestone score for programs in which the FPD is on the CCC; ^c^*p* < 0.05 compared with unweighted composite milestone score with all members on CCC

*ACGME* Accreditation Council for Graduate Medical Education, *CCC* Clinical Competency Committee, *FPD* program director, *LOS* level of supervision

### Testing hypothesis 2: Some subcompetencies would be more critical than others such that weighted scores will have a stronger correlation

**Fig. 3:**
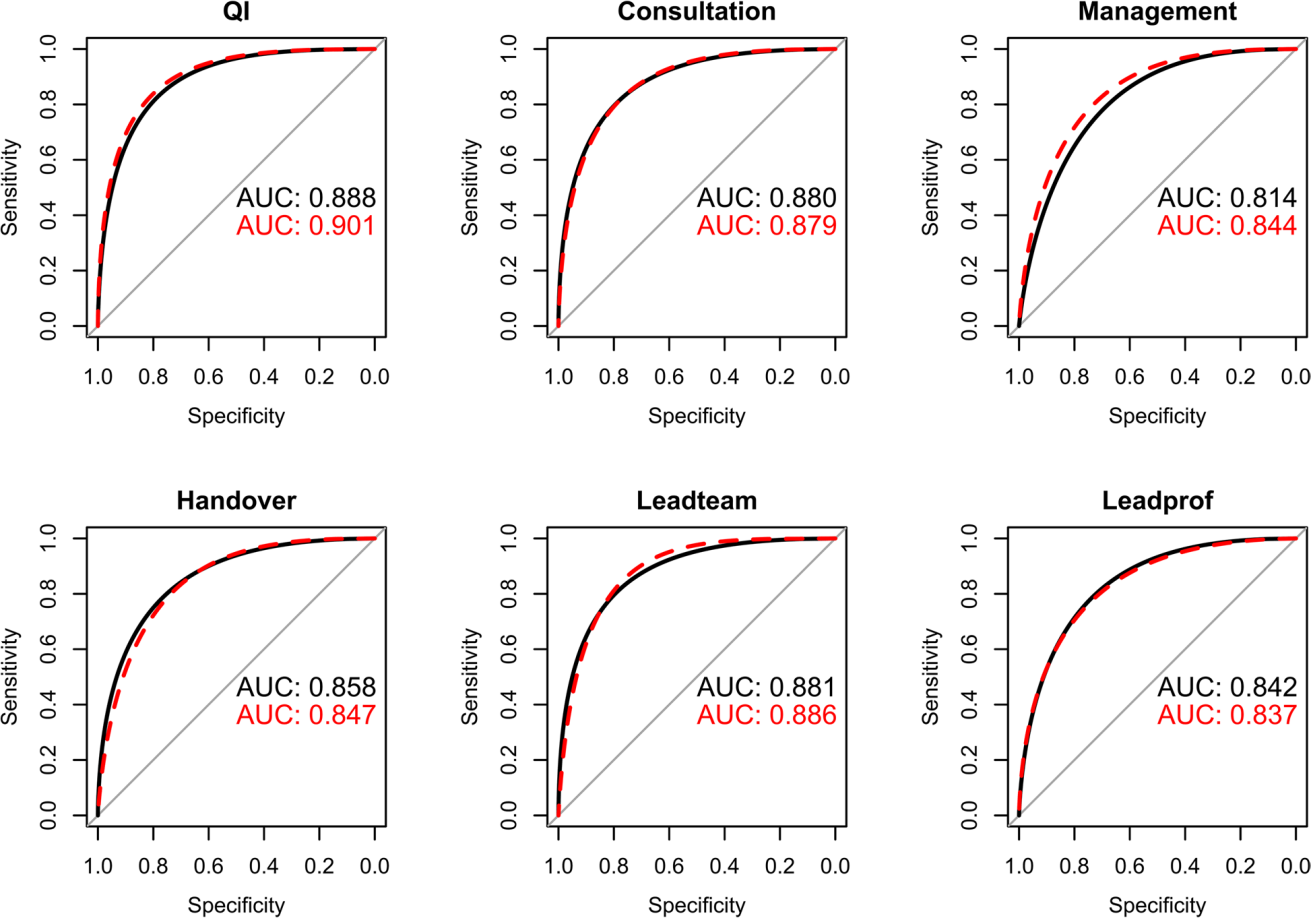
ROC curves and area under the curve for spring 2015 data using unweighted Accreditation Council for Graduate Medical Education subcompetency milestone composite score to achieve EPA level of supervision ratings of 4 or 5 (solid line; black) or only 5 (dashed line; red). Ratings utilized data from all members of the Clinical Competency Committee. Area under the curve in black is based upon a rating of 4 or 5 while that in red used a rating of 5. Abbreviations: ROC = receive operating characteristics, AUC = area under the curve, EPA = Entrustable professional activities

Correlations calculated using a weighted composite milestone score were not significantly better than those calculated using an unweighted score, counter to our second hypothesis. The most parsimonious best-fitting model was thus the unweighted ACGME-reported-milestones-only composite score. Figure 3 shows ROC curves using unweighted ACGME-reported-milestones-only composite score from the spring for the 6 EPAs to predict entrustment based upon a minimum EPA LOS of 4 or 5. The area under the curve (AUC) was excellent, ranging from 0.81 (95% CI: 0.78–0.84) for Management to 0.90 (0.86–0.94) for QI. When assuming entrustment based upon attaining a LOS of 5, the AUCs were similar to those using a minimum of level 4 (*p* > 0.05). For each EPA, there was no difference between the AUC in fall and spring (*p* > 0.05) or based upon FPD CCC membership (*p* > 0.05).

### Testing hypothesis 3: If only those milestones required for reporting to the ACGME were included in the analysis, the correlation between EPA LOS rating and average milestone level would be weaker

Goodness-of-fit for weighted models using all milestones or only those reported to the ACGME were both excellent and did not differ (*p* = 0.72), counter to our third hypothesis that the correlation using all mapped milestones would be stronger. The comparative fit index and root mean square error of approximation of models using all milestones were 0.999 (> 0.95 is excellent) and 0.034 (< 0.05 is excellent), respectively, while values using only the ACGME reported milestones were 0.998 and 0.043 [28]. Prediction equations for each model are shown in Table 4.

**Table 4:** Equations used to determine subcompetency milestone level based upon EPA level of supervision ratings and whether all mapped subcompetency milestones were used in the model or only those reported to the ACGME^a^

Subcompetency	Utilizing all 29 Subcompetency Milestones	Utilizing only 21 ACGME Reported Subcompetency Milestones
ICS1	1.1699 + 0.6598(LOS_Consultation_)	-
ICS2	1.7798 + 0.5334(LOS_Leadteam_)	-
ICS3	1.1496 + 0.1667(LOS_Leadprof_) + 0.4791(LOS_Handover_)	1.2272 + 0.2205(LOS_Leadprof_) + 0.4192(LOS_Handover_)
ICS4	1.724—0.0098(LOS_Leadprof_) + 0.5871(LOS_Leadteam_) -0.0277(LOS_Consultation_)	1.5472 + 0.0473(LOS_Leadprof_) + 0.3620(LOS_Leadteam_) + 0.1742(LOS_Consultation_)
ICS5	1.0062 + 0.6329(LOS_Consultation_)	1.0049 + 0.63332(LOS_Consultation_)
ICS6	1.4236 + 0.2803(LOS_Management_ + 0.291(LOS_Handover_)	-
MK2	1.1398 + 0.0378(LOS_Leadprof_) + 0.6143(LOS_Consultation_)	1.1530 + 0.0568(LOS_Leadprof_) + 0.5954(LOS_Consultation_)
PBLI1	1.3572 + 0.605(LOS_Consultation_)	1.3563 + 0.6052(LOS_Consultation_)
PBLI4	1.4387 + 0.636(LOS_QI_)	1.4295 + 0.6391(LOS_QI_)
PBLI5	1.0036 + 0.6473(LOS_Handover_)	-
PBLI7	1.7149 + 0.3266(LOS_QI_) + 0.2229(LOS_Handover_)	1.6741 + 0.2919(LOS_QI_) + 0.2586(LOS_Handover_)
PBLI8	1.5107 + 0.604(LOS_Leadteam_)	-
PBLI9	1.6212 + 0.5898(LOS_Leadteam_)	1.6250 + 0.5887(LOS_Leadteam_)
PC12	1.624—0.01(LOS_Leadprof_) + 0.5971(LOS_Leadteam_)	1.6256 + 0.0482(LOS_Leadprof_) + 0.5441(LOS_Leadteam_)
PC2	0.8874 + 0.7066(LOS_Consultation_)	-
PC3	0.9502 + 0.6875(LOS_Handover_)	0.9803 + 0.6800(LOS_Handover_)
PC6	1.1439 + 0.6207(LOS_Consultation_)	1.1426 + 0.6210(LOS_Consultation_)
PPD4	1.8176 + 0.0532(LOS_Leadprof_) + 0.3391(LOS_Leadteam_)	-
PPD6	1.4679—0.0286(LOS_Leadprof_) + 0.6234(LOS_Leadteam_)	1.1426—0.0272(LOS_Leadprof_) + 0.5724(LOS_Leadteam_)
PPD8	1.1983 + 0.6201(LOS_Consultation_)	1.1972 + 0.6204(LOS_Consultation_)
PROF2	2.0305 + 0.5323(LOS_Management_)	2.0249 + 0.5341(LOS_Management_)
PROF4	2.3314 + 0.3266(LOS_QI_)	-
SBP1	1.6532 + 0.5923(LOS_Management_)	1.6472 + 0.5941(LOS_Management_)
SBP2	1.3604 + 0.5652(LOS_Consultation_)	1.3596 + 0.5693(LOS_Consultation_)
SBP3	1.4241 + 0.2285(LOS_Management_) + 0.381(LOS_QI_)	1.4136 + 0.3005(LOS_Management_) + 0.3082(LOS_QI_)
SBP4	1.501 + 0.0874(LOS_Leadprof_) + 0.5383(LOS_QI_)	-
SBP5	2.0918 + 0.021(LOS_Management_) + 0.3295(LOS_Leadteam_) + 0.0571(LOS_QI_)	2.0879 + 0.0364(LOS_Management_) + 0.2791(LOS_Leadteam_) + 0.0994(LOS_QI_)
SBP6	1.7567 + 0.1286(LOS_Managment_) + 0.4518(LOS_QI_)	1.7454 + 0.2224(LOS_Management_) + 0.3562(LOS_QI_)
SBP7	1.6227 + 0.095(LOS_Leadprof_) + 0.3355(LOS_QI_)	-

*ACGME* Accreditation Council for Graduate Medical Education, *LOS* level of supervision

^a^Subcompetencies are those associated with the Pediatric Milestones; [42] subscripts indicate specific EPA

The external validation sample included 1373 EPA LOS ratings from 503 (36.6%) first year, 448 (32.6%) second year and 422 (30.7%) third year fellows. The comparative fit index and root mean square error of approximation of models using the ACGME prediction equations were 0.994 (> 0.95 is excellent) and 0.071 (0.06–0.08 is fair), respectively [28].

## Discussion

In support of our first hypothesis, we found a strong relationship between milestone levels for subcompetencies mapped to an EPA and the LOS ratings for that EPA, providing validity evidence for both approaches. Our data do not support the second hypothesis in that we found the relationship between EPA LOS and milestone scores was nearly identical whether we used the unweighted or weighted milestone scores. Likewise, the relationship between milestone level and EPA LOS rating was similar when only the ACGME reported milestones were utilized in the model compared with using milestones from all 29 subcompetencies mapped to the six EPAs.

Our results are similar to those of Larrabee et al., who examined the association between 27 EPAs that they developed for 4 core rotations in pediatric residency with milestones mapped to these EPAs [29]. They found a strong correlation between the two ratings, with an overall median R2 of 0.81. Although these investigators focused on residents and used a different LOS scale, the concordance between Larrabee’s findings and ours nevertheless suggests that the relationship between milestones and EPA LOS is generalizable and not dependent upon a particular group of trainees or a specific LOS scale.

The areas under the curve for all EPAs were very high. Except for one, this was irrespective of whether entrustment was set at level 5 or at level 4 or 5, indicating that the relationship was not solely dependent upon how entrustment was defined. While executing the EPA without supervision is the goal, not all FPDs believe that fellows must achieve level 5 (unsupervised practice) in all EPAs to graduate, indicating that some supervision may still be needed [30–32]. Also, for some EPAs, while the correlations were somewhat weaker if the FPD was not a member on the CCC, the differences were small and the AUCs for the ROC curves were not affected.

We constructed equations to predict milestone level based upon EPA LOS rating that had an excellent goodness of fit. In both the derivation and validation samples, the comparative fit index was excellent. While there was a slight decline in the root mean square error of approximation using the data 4 years later, coupled with the strong comparative fix index, the overall goodness-of-fit was still very good. Since the spring 2019 data represent assessments of different fellows, and likely include CCCs that had differing compositions, this shows that the equations maintained their precision over time.

Showing that there is a strong relationship between milestones and EPA LOS helps to address FPD’s concerns about the additional work involved as EPAs become a required element of trainee assessment [33–35]. Faculty find using EPA LOS scales very intuitive and generating milestone levels based on EPA LOS ratings should be timesaving [33]. These predicted milestone ratings can serve as a starting point when the CCC discusses each trainee and makes the final assignments. We used milestones version 1.0 in our study to develop the equations, but milestones 2.0 will shortly be implemented across all specialties and subspecialties. Our finding that models using all milestones compared with only those reported to the ACGME are similar will make it easier to revise the equations with updated milestones.

We found little difference in the correlations between EPA LOS and milestones based on whether or not the FPD was a member of the CCC. These findings are consistent with our previous report that the association between FPD and CCC assignment of EPA LOS is strong [24]. With the exception of the Management EPA in the fall, the correlations for when the FPD made the assessments independently of the CCC were also similar. It is reassuring that the relationship between LOS and milestones is not affected by FPD membership on the CCC.

While both approaches to assessment are highly related, it is important to recognize the contribution of each in creating an overall program of trainee assessment [17, 36–38]. As program directors and CCCs make decisions about trainee progression toward unsupervised practice, the need to focus more on either EPA LOS ratings or milestone levels may depend on the circumstances. For high performing trainees who require minimal supervision to execute an EPA, milestone levels may be less important than for a trainee who is early in development and requires more supervision. In the latter case, the descriptive language of the skills included in each milestone level can help the trainee focus on improvements needed to effectively perform the EPA, especially if they are below the normative national standards or not meeting program expectations [39–41].

There are several limitations to this study. We asked FPDs to assign milestones before assigning LOS for the EPAs rather than randomizing the assessments. This could have biased their rating for EPA LOS. The initial data collection period was the first time FPDs had to report milestones to the ACGME and assign EPA LOS ratings. With more experience, the application of these assessments may change, although we saw no difference in results between the two reporting periods. In addition, the goodness-of-fit of the equations using the ACGME-reported milestones suggests that there has not been much change in in the milestone-LOS relationship over time. Finally, we used the Common Pediatric Subspecialty EPAs, and the findings cannot necessarily be extrapolated to assessments made using EPAs developed by other specialties.

## Conclusions

We found strong agreement between assessments based on subcompetency milestones with those using EPA LOS but no difference if the subcompetencies were weighted or if only the ACGME reported subcompetencies were used. In addition, these data provide additional validity evidence for both types of assessment. We were also able to develop equations to generate milestone levels based on EPA supervision ratings using the ACGME reported subcompetencies. This will help to address the time burden faced by educators while also allowing them the flexibility to use EPAs and milestones as appropriate in assessing their trainees and “developing “a program of assessment fit for purpose” [17]. 

## Data Availability

The datasets used and/or analyzed during the current study are available from the corresponding author on reasonable request.

## Abbreviations

ACGME: Accreditation Council for Graduate Medical Education
APPD LEARN: Association of Pediatric Program Directors Longitudinal Educational Assessment Research Network
AUC: Area under the curve
CCC: Clinical Competency Committee
Consultation EPA: Provide consultation to other healthcare providers caring for children and adolescents and refer patients requiring further consultation to other subspecialty providers if necessary
EPA: Entrustable Professional Activities
FPD: Fellowship program director
Handover: EPA: Facilitate handovers to another healthcare provider either within or across settings
ICS: Core competency: Interpersonal and communication skills
Leadteam: EPA: Lead an interprofessional health care team
Leadprof: EPA: Lead within the subspecialty profession
LOS: Level of supervision
Management: EPA: Contribute to the fiscally sound, equitable and collaborative management of a healthcare workplace
PBLI: Core competency: Practice-based learning and improvement
PC: Core competency: Patient care
PPD: Core competency in Pediatrics: Personal and Professional Development
QI: EPA: Apply public health principles and quality improvement methods to improve population health
ROC: Receiver operating characteristic
SBP: Core competency: System-based practice
